# *Bifidobacterium lactis* BB-12 Attenuates Macrophage Aging Induced by D-Galactose and Promotes M2 Macrophage Polarization

**DOI:** 10.1155/2019/4657928

**Published:** 2019-12-19

**Authors:** Da-yong Zhang, Zheng-yang Pan, Xiong-kai Yu, Yi-fan Chen, Chen-hao Gao, Yu-tian Yang, Xue-fan Jiang, Na Li, Jian-ping Pan

**Affiliations:** ^1^Department of Clinical Medicine, School of Medicine, Zhejiang University City College, Hangzhou 310015, China; ^2^Department of Otorhinolaryngology, Zhejiang Provincial People's Hospital, Hangzhou, Zhejiang 310014, China; ^3^People's Hospital of Hangzhou Medical College, Hangzhou, Zhejiang 310014, China

## Abstract

Immunosenescence comprises a set of dynamic changes occurring in innate and adaptive immune systems, and macrophage aging plays an important role in innate and adaptive immunosenescence. However, function and polarization changes in aging macrophages have not been fully evaluated, and no effective method for delaying macrophage senescence is currently available. The results of this study reveal that D-galactose (D-gal) can promote J774A.1 macrophage senescence and induce macrophage M1 polarization differentiation. *Bifidobacterium lactis* BB-12 can significantly inhibit J774A.1 macrophage senescence induced by D-gal. IL-6 and IL-12 levels in the BB-12 groups remarkably decreased compared with that in the D-gal group, and the M2 marker, IL-10, and Arg-1 mRNA levels increased in the BB-12 group. BB-12 inhibited the expression of p-signal transducer and activator of transcription 1 (STAT1) and promoted p-STAT6 expression. In summary, the present study indicates that BB-12 can attenuate the J774A.1 macrophage senescence and induce M2 macrophage polarization, thereby indicating the potential of BB-12 to slow down immunosenescence and inflamm-aging.

## 1. Introduction

As we age, changes in essentially all physiological functions, including immunity, become apparent [1]. Immunosenescence is the state of dysregulated immune function that contributes to the increased incidence of different chronic diseases, such as infections, autoimmune disorders, chronic inflammatory diseases, and cancer [2, 3]. Macrophage, which is one of the important immune cells, plays a pivotal role in the regulation of the innate and acquired immune responses [4]. However, the present study showed that macrophages also experience changes due to aging and have an immense influence on immunosenescence [5]. The quantity of aging-related galactosidase- (SA-*β*-gal-) positive macrophages in the senescence accelerated-prone mice (SAPM8) is considerably larger than that in young mice, and CD36 expression levels and IL-10 level decrease in aging macrophages [6]. Downregulated p16 (an aging promoting factor) can significantly delay macrophage aging and upregulate Arg-1 [7], MRC1, and AMAC1 expression levels [8]. These results indicated that macrophages also undergo changes due to aging, and functional change in aging macrophages may be an important constituent part of immunosenescence and inflamm-aging. However, the study of aging macrophage is still insufficient, and a stable, simple, and convenient in vitro induction method for macrophage aging remains unavailable. D-Galactose (D-gal) is a ROS accelerant that is a common reagent used to build an aging animal model. Our group [9, 10] and another research group [11] have found that D-gal is also a favorable inducer for the cell aging model in in vitro research. Therefore, in the present study, the influence of D-gal on macrophage aging and polarization was observed first.

As a representative of intestinal probiotics, Bifidobacterium is widely used in nutritional supplements, has multiple physiological activities, and facilitates an organism's health [12]. However, the quantity of Bifidobacteria in aging individuals considerably decreases due to weakened intestinal barrier function, decreased gastric acid secretion, and reduced immunologic functions with age [13–15]. The quantity of intestinal Bifidobacteria in healthy longevity of older people in Bama County is considerably higher than that of normal people [16]. The lipoteichoic acid in Bifidobacterium can also delay cell senescence [17]. Hence, Bifidobacterium has a positive influence on an organism or cellular aging. Bifidobacterium BB-12 (BB-12) is a catalase-negative, rod-shaped Bifidobacterium derived from animals. BB-12 was deposited in the cell culture bank of Chr. Hansen in 1983, and one of *Bifidobacterium* spp. has been studied and commercially used to the greatest extent [18]. At present, BB-12 is classified as *Bifidobacterium animalis* subsp. *lactis*. BB-12 can exert multiple health-promoting effects on an organism [19, 20] and is beneficial to immune function [21–23]. Bifidobacterium supernatant contains a variety of active substances, including polysaccharides [24], proteins [25], and fatty acids [26]. These ingredients have antioxidation, immune regulation, and other antiaging effects [25, 27, 28]. Bifidobacterium supernatant can also reverse D-gal-induced organ aging and decrease memory in aging rats [29]. However, the role of BB-12 on cellular aging is still poorly understood. In the present study, the effects of BB-12 supernatant on D-gal-induced macrophage aging were observed for the first time. This observation provided fundamental experimental data regarding effective methods for delaying macrophage aging through the application of Bifidobacterium.

Heterogeneity and functional plasticity are two of the most important characteristics of macrophages. Under the effects of different stimulants (pathogenic microorganism products, damaged cells, and activated lymphocytes) in the cellular microenvironment, macrophages can be polarized into two major types, that is, classically (M1 macrophage) and alternatively activated macrophages (M2 macrophage) [30, 31]. The sterilizing functions of M1 macrophages, productions of proinflammatory factors (IL-6, IL-12, etc.), and complement-mediated phagocytic functions can be significantly upregulated to exert the anti-infection effect [32]. M2 macrophages secrete anti-inflammatory cytokines (e.g., IL-4 and IL-10) and exert effects on tissue repair and reconstruction processes [33]. The study showed that M1 macrophage levels in mice increase with age, and this increase is related to the declined damage repair ability of aging mice and long-term maintenance of an inflammation state [34]. The expression levels of macrophage M2 polarization marker IL-10 in SAPM8 mice decreased [6], and delaying microphage aging can upregulate the expression level of macrophage M2 polarization marker Arg-1 [7]. These results indicated that after macrophage aging, they can be differentiated into proinflammatory M1 state. Thus, macrophage aging may be closely related to the fact that multiple organs in aging individual are under chronic and sustainable inflammation state for a long time, which is also called inflamm-aging. In the present study, the effects of BB-12 on macrophage polarity and aging were further observed. The molecular mechanisms that determine M1 or M2 polarization involve specific transcription factors, such as signal transducer and activator of transcription 1 (STAT1), which is a critical transcription factor and results in M1 polarization [35, 36]. Meanwhile, STAT6 is an essential protein in activating M2 macrophage and regulating the balance of inflammatory response [37]. STAT1 or STAT6 is also closely linked to elderly degenerative diseases and immunosenescence. In Alzheimer's disease, STAT1 and senescence-related protein p53 are considerably upregulated in microglia [38]. Impaired actin filament dynamics, which is a common phenomenon in cellular senescence and degenerative glial cells, can reduce phospho-STAT6 levels in microglia [39]. Therefore, to explore the mechanisms of BB-12 regulating the polarity of macrophages, we further examined the effects of BB-12 on the phosphorylation of STAT1/STAT6 in macrophages.

Immunosenescence is an important factor influencing organism aging. In this study, D-gal was utilized to construct an in vitro macrophage aging research model, the influences of BB-12 on macrophage aging and polarity were examined, and its molecular mechanism was explored.

## 2. Methods and Materials

### 2.1. Bacterial Culture and Bifidobacterium Supernatants

*Bifidobacterium animalis* subsp. *lactis* BB-12 were purchased from Chr. Hansen, Denmark. BB-12 was incubated in bacteria culture medium (MRS) broth (Difco, Detroit, MI, USA) supplemented with 5% (*W*/*V*) galactose in the anaerobic conditions at 37°C for 24 h, followed by dilution in the MRS broth and incubation to reach the exponential phase with the density of 0.5 at an optical density (OD) of 600 nm. The culture suspensions were centrifuged at 5,000 × *g* for 10 min at 4°C, then filter-sterilized through 0.22 *μ*m filters. Finally, the supernatant was standardized to 10 *μ*g of protein/*μ*L using the BCA protein assay kit (Beyotime, China).

### 2.2. J774A.1 Cell Culture and Treatment

Mouse monocyte-macrophage cells (J774A.1) were obtained from the Shanghai Institute of Cell Biology at Chinese Academy of Sciences. The cells were cultured and maintained in Roswell Park Memorial Institute (RPMI-1640) supplemented with streptomycin (100 mg/mL), penicillin (100 U/mL), and 10% fetal bovine serum (FBS) in a 5% CO_2_ incubator at a temperature of 37°C. Then, J774A.1 cells were divided into the following groups: (1) Control group: macrophages were cultured for 24 h in RPMI-1640 containing 10% FBS. (2) D-gal treatment groups: J774A.1 cells were incubated for 24 h in RPMI-1640 containing 10% FBS in the presence of 0.1, 1, or 10 g/L D-gal (Sigma, USA), respectively. (3) BB-12 treatment groups: briefly, J774A.1 cells were treated with the BB-12 supernatant (100 *μ*g/mL) in RPMI-1640 containing 10% FBS for 30 min,1 h, or 6 h, then culture medium were changed, and the cells were further cultured in RPMI-1640 containing 10% FBS in the presence 10 g/L D-gal for 24 h.

### 2.3. SA-*β*-Gal Staining

Senescence-associated *β*-galactosidase (SA-*β*-gal) staining was performed using a SA-*β*-gal staining kit (Beyotime, China) following the manufacturer's protocol. The treatment methods for the J774A.1 in each group were the same as described above. The cells were fixed in 4% (*v*/*v*) formaldehyde for 5 min and then were stained with SA-*β*-gal staining solution at pH 6.0 for 24 h. The SA-*β*-gal-positive cells exhibited a blue color. The number of positive cells was counted under a phase-contrast microscope. The experiment was repeated five times in each group.

### 2.4. ROS Staining

ROS staining was performed using an ROS staining kit (Beyotime, China) following the manufacturer's protocol. After each group was cultured according to the above treatment methods, the cells were washed three times in PBS and incubated in ROS staining solution (DCFH-DA) at 37°C for 20 min. After washing, the nuclei were counterstained with Hoechst 33342 (Sigma, USA). The cells were observed using a fluorescence microscope. To quantify the ROS level, the DCFH fluorescence intensity in the cells was detected by a flow cytometer (Calibur, BD Biosciences, USA) at an excitation wavelength of 488 nm and an emission wavelength of 525 nm. Experiments were repeated three times.

### 2.5. Detection of Cytokines by Enzyme-Linked Immunosorbent Assay (ELISA)

The J774A.1 macrophages were cultured as described above. Supernatants of J774A.1 cell culture were collected after 6 h of incubation. Concentrations of IL-6, IL-12, and IL-10 were determined by ELISA according to the manufacturer's instructions (Peprotech, Mexico) in triplicate wells for all individual treatments.

### 2.6. Detection of Cell Viability by the Cell Counting Kit -8 (CCK-8)

The J774A.1 macrophages were plated and treated as described above in 96-well plates (three wells per group). Approximately 10 *μ*L of CCK-8 (Dojindo, Japan) was added to the culture medium, and the OD value of the cells was measured at 450 nm using an ELISA reader (Thermo Multiskan GO, USA) according to the manufacturer's instructions. The experiment was repeated 3 times, and the average values were obtained.

### 2.7. RNA Extraction and Quantitative Real-Time PCR (qPCR)

RNA was isolated from J774A.1 macrophages using TRIzol according to the manufacturer's recommendation (Invitrogen, USA). The RNA was quantified, and its quality was assessed by agarose gel electrophoresis and absorbance measurements at *λ*260/*λ*280 nm with the Nanodrop ND-1000 spectrophotometer. First-strand cDNA was synthesized using RevertAid first strand cDNA synthesis kit (Takara, Japan), according to the manufacturer's protocol. The reverse transcription reactions were performed at 25°C for 5 min, followed by 42°C for 60 min and 70°C for 5 min. The cDNAs were stored at −80°C for later use. qPCR was performed using a CFX-96 Real-time PCR Detection System (Bio-Rad, USA). SYBR Green Master Mix (Bio-Rad, USA) was used to examine relative gene expression. Glyceraldehyde 3-phosphate dehydrogenase (GAPDH) was used to normalize the measured transcript, and the samples were run in triplicate. The relative quantity of gene expression was calculated automatically by the 2−*ΔΔ*Cq method. Primers were synthesized by Shanghai Shenggong Biology Engineering Technology Service, Ltd. (Shenggong, China), as follows: Arg-1 forward, 5′-AGACCACAGTCTGGCAGTTG-3′ and reverse, 5′-CCACCCAAATGACACATAGG-3′; GAPDH forward, 5′-CTGCACCACCAACTGCTTAG-3′ and reverse 5′-GTCTGGGATGGAAATTGTGA-3′.

### 2.8. Western Blot Analysis

To assay the expression of p53, p16, STAT1, STAT6, p-STAT1, and p-STAT6, the total cellular protein was extracted through the following methods: The different J774A.1 cell treatment groups were washed in cold-buffered PBS and were then lysed in RIPA buffer (150 mM NaCl, 1% Triton X-100, 0.5% NaDOD, 0.1% SDS, and 50 mM Tris, pH 8.0). After centrifugation (12,000 rpm, 5 min) at 4°C, the protein supernate was transferred into new tubes. The protein concentration of the samples was determined with a bicinchoninic acid protein assay (Pierce, USA). A 40-lg sample of the total protein was resolved using 12.5% SDS-PAGE and transferred onto polyvinylidene difluoride (PVDF, Millipore, USA) membranes. The membranes were blocked with 5% nonfat milk at room temperature for 1 h in Tris-buffered saline containing Tween 20 (TBST). Primary antibodies to detect p53 (1 : 1,000, BD, USA), p16 (1 : 1000, Santa Cruz, USA), STAT1 (1 : 1,000, CST, USA), STAT6 (1 : 1,000, CST, USA), p-STAT1 (Tyr701, 1 : 1,000, Thermo Fisher Scientific, USA), p-STAT6 (Thr645, 1 : 1,000, Thermo Fisher Scientific, USA), and *β*-actin (1 : 5000, BD, USA) were incubated overnight with the membranes at 4°C. Membranes were incubated with horseradish peroxidase- (HRP-) conjugated anti-rabbit secondary antibodies (1 : 2000, Dako, USA), and proteins were detected by enhanced chemiluminescence (ECL) (Amersham Biosciences Corp, USA). *β*-Actin was used as the internal control to normalize the loading materials. The p-STAT1 and p-STAT6 levels were quantified using ImageJ software (NIH, USA) and were expressed as the ratio after normalization to the total STAT1 and total STAT6.

### 2.9. Statistical Analysis

All experiments were performed at least in triplicate. All data are presented as the mean ± standard deviation of the mean (SD). Significance testing was performed using one-way analysis of variance (ANOVA) to compare data from different experimental groups, and multiple comparisons were made by Bonferroni's test. For all analyses, A *P* value < 0.05 was considered significant.

## 3. Results

### 3.1. D-Gal Promoted J774A.1 Macrophage Senescence

**Figure 1:**
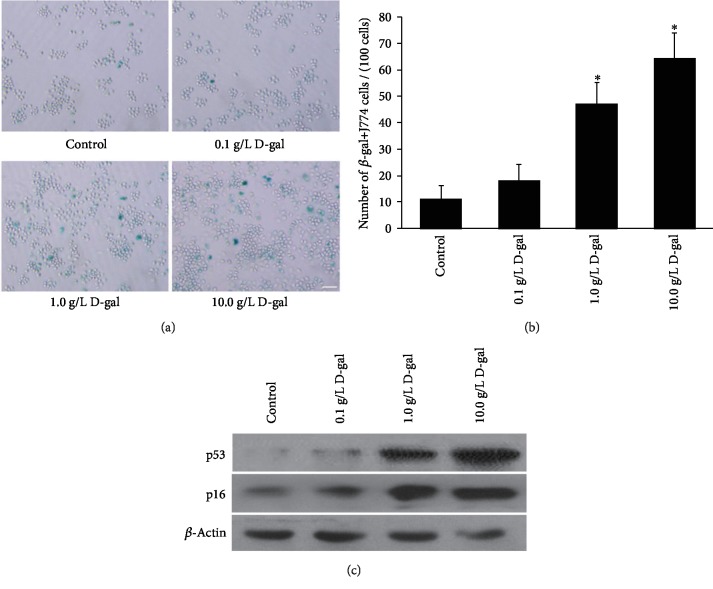
Effects of D-gal on J774A.1 macrophage senescence. (a) SA-*β*-gal staining. With the increase in D-gal concentration, the number of SA-*β*-gal-positive cells increases in the 1 and 10 g/L D-gal group. Scale bar = 50 *μ*m. (b) SA-*β*-gal staining cell quantification. The total number of SA-*β*-gal-positive cells among 100 random cells was determined through phase-contrast microscopy. The results showed that the number of SA-*β*-gal-positive J774A.1 cells/100 cells in the 1.0 and 10.0 D-gal groups remarkably increased compared with that in the control group (∗*P* < 0.01, *n* = 5). (c) Western blot analysis of p53 and p16 expression levels. The p53 and p16 expression levels were gradually higher in the 0.1, 1.0, and 10.0 g/L D-gal groups compared with those in the control group. *β*-Actin was used as the internal control.

SA-*β*-gal staining was used to observe the effect of D-gal on J774A.1 macrophage senescence. After culturing in different D-gal concentrations (0.1, 1.0, or 10.0 g/L) for 24 h, the number of SA-*β*-gal-positive J774A.1 cells and the staining intensity of SA-*β*-gal increased (Figure 1(a)). The cell count revealed that the number of SA-*β*-gal-positive cells compared with that of the control group (11.2 ± 5.0/100 cells) significantly increased in the 1.0 and 10.0 g/L D-gal group (47.2 ± 8.1 and 64.6 ± 9.4/100 cells, *P* < 0.01; Figure 1(b)). To evaluate the effects of D-gal on the macrophage senescence further, we harvested primary macrophages from the peritonium of Sprague-Dawley (SD) rats. The results of SA-*β*-gal staining showed that 1.0 or 10.0 g/L D-gal can induce primary macrophage senescence, and the number of SA-*β*-gal-positive cells in 1.0 and 10.0 g/L D-gal groups significantly increased compared with that in the control group ([Supplementary-material supplementary-material-1]).

The results of our previous study indicated that p53 and p16 were two important mediators of cellular senescence [40]. To explore the effect of D-gal on J774A.1 macrophage senescence, we examined the expression of senescence-related proteins p53 and p16 in J774A.1 macrophage. The Western blot results showed that with the increase in D-gal concentration, the p53 and p16 expression levels in the D-gal groups increased compared with those in the control group (Figure 1(c)). These results suggested that D-gal can induce macrophage senescence, and 10 g/L was a suitable D-gal-induced macrophage senescence concentration in vitro.

### 3.2. D-Gal Inhibited J774A.1 Macrophage Proliferation

**Figure 2:**
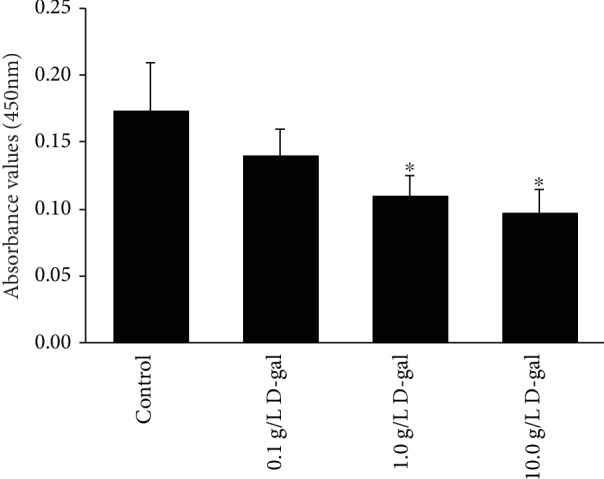
Effect of D-gal on J774A.1 proliferation. The CCK8 result showed that the absorbance value significantly decreased in 1.0 and 10.0 g/L D-gal groups compared with the control group (∗*P* < 0.05, *n* = 3).

Meanwhile, the cell proliferation was examined by CCK-8. The results indicated that J774A.1 macrophage proliferation can be inhibited by D-gal, and the absorbance values in 1.0 and 10.0 g/L D-gal groups (0.11 ± 0.01 and 0.10 ± 0.02) significantly decreased compared with those in the control group (0.17 ± 0.04, *P* < 0.05; Figure 2). The result indicated that D-gal can inhibit J774A.1 macrophage proliferation.

### 3.3. D-gal Induced ROS Generation in J774A.1 Macrophage

**Figure 3:**
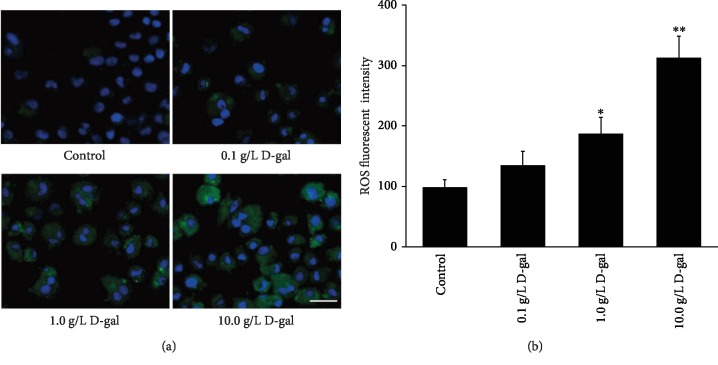
Effects of D-gal on ROS generation in J774A.1 cell. (a) ROS staining. In the 1.0 and 10.0 g/L D-gal groups, increased ROS-stained cells were observed under a fluorescence microscope compared with that in the control group. Green, ROS staining; blue, Hoechst 33342 staining. Scale bar = 25 *μ*m. (b) ROS level quantification. The DCFH fluorescence intensity in the 1.0 and 10.0 g/L D-gal groups was significantly increased compared that of the with the control group (^∗^*P* < 0.05, ^∗∗^*P* < 0.01; *n* = 3).

ROS staining showed that the number of ROS-stained cells and the DCFH fluorescent level of the cells gradually increased with the increase in D-gal concentration (Figure 3(a)). The quantification of the DCFH fluorescence intensity showed that the intensity of DCFH fluorescence in 1.0 and 10.0 g/L D-gal groups (186.6 ± 28.4 and 312.9 ± 35.6) significantly decreased compared that in the control group (97.6 ± 13.8, *P* < 0.05 or *P* < 0.01; Figure 3(b)). These results indicated that D-gal can promote the ROS production in J774A.1 cell.

### 3.4. Effects of D-Gal on Polarity of J774A.1 Macrophages

**Figure 4:**
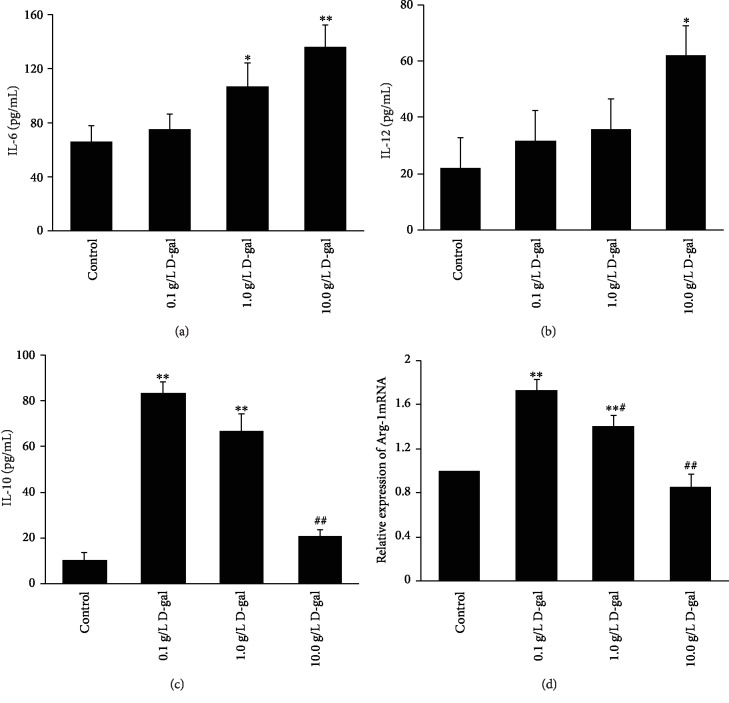
Effects of D-gal on polarity of J774A.1 macrophages. The IL-6 (a), IL-12 (b), and IL-10 (c) levels were assayed by ELISA. The results showed that the IL-6 concentrations in the 1.0 and 10.0 g/L D-gal groups were significantly higher than those in the control group (^∗^*P* < 0.05, ^∗∗^*P* < 0.01, *n* = 3). The IL-12 concentrations in the 10.0 g/L D-gal group increased compared with those in the control group (^∗^*P* < 0.01, *n* = 3). The IL-10 level increased in the 0.1, 1.0, and 10.0 g/L D-gal groups compared with that in the control group (^∗^*P* < 0.05, ^∗∗^*P* < 0.01, *n* = 3). However, the IL-10 level in the 10.0 g/L D-gal group significantly decreased compared with that in the 0.1 g/L D-gal group (^##^*P* < 0.01, *n* = 3). (d) qPCR analysis of the relative expression level of Arg-1 mRNA. GAPDH was used as an internal control. The Arg-1 mRNA expression level in the 1.0 and 10.0 g/L D-gal groups was gradually lower than that in the 0.1 g/L D-gal group (^#^*P* < 0.05, ^##^*P* < 0.01, *n* = 3).

M1 macrophages can secrete a large amount of the proinflammatory markers IL-6 and IL-12, while M2 macrophages release additional anti-inflammation marker IL-10 and have a high Arg-1 expression level. We examined the IL-6, IL-12, and IL-10 levels by ELISA and the Arg-1 mRNA expression by qPCR. Compared with the control group (IL-6: 66.2 ± 12.0 pg/mL, IL-12: 21.9 ± 10.8 pg/mL), the IL-6 level was evidently higher in the 1.0 and 10.0 g/L D-gal groups (106.8 ± 17.4 and 135.9 ± 16.0 pg/mL, *P* < 0.05, or *P* < 0.01; Figure 4(a)), and the IL-12 level was higher in the 10.0 g/L D-gal group (61.9 ± 10.7 pg/mL, *P* < 0.05; Figure 4(b)). The result suggested that D-gal can promote M1 macrophage polarization.

The ELISA results showed that with the increase in D-gal concentration, the IL-10 level gradually decreased in D-gal treatment groups. The IL-10 level in the 10.0 g/L D-gal group (20.8 ± 2.6 pg/mL) significantly decreased compared with than that in the 0.1 g/L D-gal group (*P* < 0.01, Figure 4(c)). The Arg-1 mRNA expression level also decreased in the 1.0 and 10.0 D-gal groups than that in the 0.1 g/L D-gal group (Figure 4(d)). These results indicated that M2 macrophage polarization was inhibited in the high D-gal concentration group compared with that in the low D-gal concentration group.

### 3.5. BB-12 Can Inhibit J774A.1 Macrophage Senescence Induced by D-Gal

**Figure 5:**
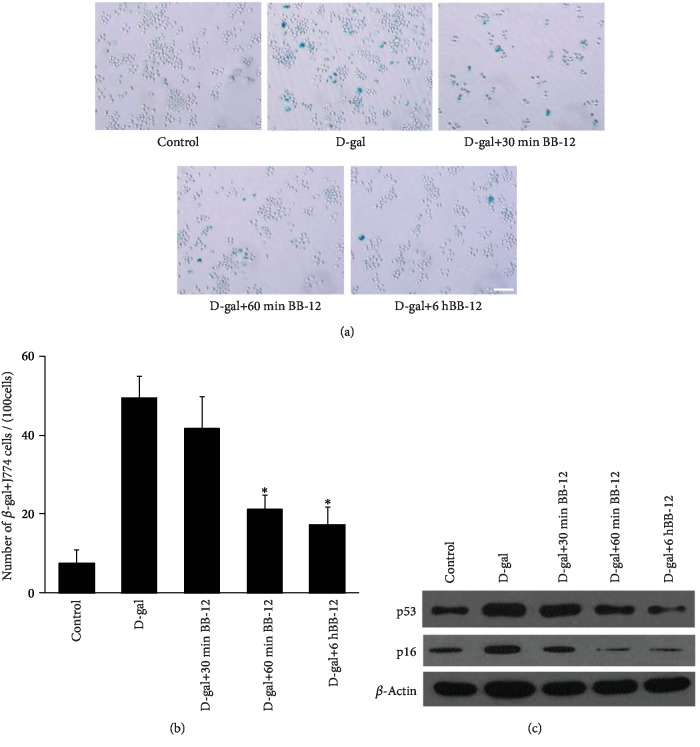
Effects of BB-12 on J774A.1 macrophage senescence. (a) SA-*β*-gal staining. Compared with the D-gal group, the SA-*β*-gal-positive cells in the BB-12 group significantly decreased. (b) SA-*β*-gal-positive cell quantification. The counts showed that the number of SA-*β*-gal-positive cells in the D-gal+60 min BB-12 and D-gal+6 h BB-12 groups decreased significantly compared with that in the D-gal group (^∗^*P* < 0.01, *n* = 5). (c) Western blot analysis of the p53 and p16 expression levels. The p53 and p16 expression levels were gradually lower in the D-gal+30 min BB-12, D-gal+60 min BB-12, and D-gal+6 h BB-12 groups compared with those in the D-gal group. *β*-Actin was used as the internal control.

As shown in Figure 5(a), after treatment with BB-12 supplement, the SA-*β*-gal-positive J774A.1 cells gradually decreased compared with the D-gal group. The number of SA-*β*-gal-positive cells in the D-gal+60 min BB-12 (21.2 ± 3.70/100 cells) and the D-gal+6 h BB-12 groups (17.4 ± 4.28/100 cells) decreased significantly compared with that in the D-gal group (49.4 ± 5.41/100 cells, *P* < 0.01, Figure 5(b)). BB-12 treatment can also remarkably reverse the induced role of D-gal on primary macrophage senescence ([Supplementary-material supplementary-material-1]). These results suggested that Bifidobacteria BB-12 can attenuate the effect of D-gal-induced macrophage senescence.

To investigate the effects of BB-12 on the macrophage aging induced by D-gal further, we examined p53 and p16 expression levels by Western blot. The result showed that the p53 and p16 expression levels decreased in the D-gal+30 min BB-12, D-gal+60 min BB-12, and D-gal+6 h BB-12 groups compared with that in the D-gal group (Figure 5(c)).

### 3.6. BB-12 Led to M1-M2 Transition of Macrophages

**Figure 6:**
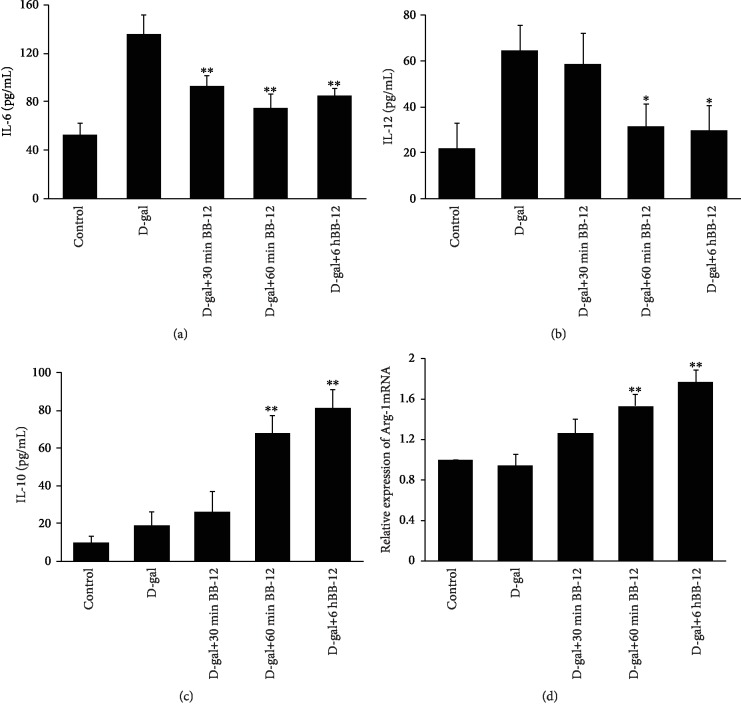
Effects of BB-12 on J774A.1 macrophage polarization. The production of IL-6 (a), IL-12 (b), and IL-10 (c) was assayed by ELISA. The results showed that the IL-6 and IL-12 levels in the D-gal+60 min and D-gal+6 h groups decreased compared with that in the D-gal group (^∗^*P* < 0.05 or ^∗∗^*P* < 0.01, *n* = 3). IL-10 in the D-gal+60 min and D-gal+6 h groups was significantly higher than that in the D-gal group (^∗∗^*P* < 0.01, *n* = 3). (d) qPCR analysis of the relative expression level of Arg-1 mRNA. GAPDH was used as an internal control. The Arg-1 mRNA expression level in D-gal+60 min and D-gal+6 h groups increased compared with that in the D-gal group (^∗∗^*P* < 0.01, *n* = 3).

To explore the effects of BB-12 on the macrophage polarization, we determined the IL-6, IL-10, and IL-12 expression levels by ELISA after J774A.1 cells or primary macrophage treated by the different times of BB-12. In J774A.1 cells, we observed significantly decreased IL-6 and IL-12 expression levels in the D-gal+60 min BB-12 (IL-6: 74.9 ± 11.3 pg/mL, IL-12: 31.6 ± 9.5 pg/mL) and D-gal+6 h BB-12 groups (IL-6: 84.9 ± 6.4 pg/mL, IL-12: 29.6 ± 11.0 pg/mL) compared with that in the D-gal group (IL-6 : 135.9 ± 16.0 pg/mL, IL-12: 64.6 ± 10.8 pg/mL, *P* < 0.05 or *P* < 0.01; Figures 6(a) and 6(b)). By contrast, the IL-10 level remarkably increased in the D-gal+60 min BB-12 (67.8 ± 9.6 pg/mL) and D-gal+6 h BB-12 groups (81.2 ± 10.0 pg/mL) compared with that in the D-gal group (19.2 ± 7.0 pg/mL, *P* < 0.01; Figure 6(c)). Similar ELISA results were also observed in primary macrophage treated with BB-12 ([Supplementary-material supplementary-material-1]). The Arg-1 mRNA expression level of J774A.1 cells in the D-gal+60 min and D-gal+6 h groups was also higher than that in the D-gal group (*P* < 0.01, Figure 6(d)). These results proved that BB-12 can lead to a shift in macrophage cells from the M1-polarized state to M2-polarized state.

### 3.7. BB-12 Promoted p-STAT6 Expression Level in J774A.1 Macrophage

**Figure 7:**
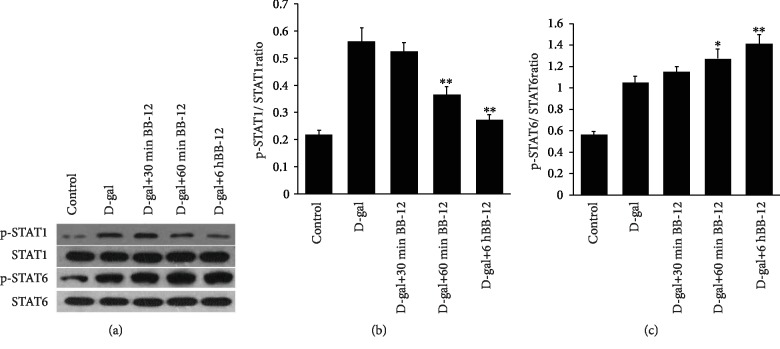
Effects of BB-12 on STAT1 and STAT6 expression levels. (a) Western blot analysis of p-STAT1, STAT1, p-STAT6, and STAT6 protein expression levels. p-STAT1 expression decreased in the group after treatment with BB-12 compared with that in D-gal group. However, with the increase in BB-12 treatment time, the p-STAT6 expression levels were gradually higher in the D-gal+30 min BB-12, D-gal+60 min BB-12, and D-gal+6 h BB-12 groups compared with those in the D-gal group. *β*-Actin was used as the internal control. (b) p-STAT1/STAT1 ratios and (c) p-STAT6/STAT6 ratios. ^∗^*P* < 0.05 or ^∗∗^*P* < 0.01 vs. the D-gal group; *n* = 3.

STAT1 and STAT6 were the key-mediated factors of M1 and M2 macrophage polarity, and the Western blot results showed that the p-STAT1 expression and p-STAT1/STAT1 ratios in the BB-12 treatment group decreased compared with those in the D-gal group. With the prolongation of the BB-12 treatment time, the p-STAT6 expression level and p-STAT6/STAT6 ratios significantly increased in the BB-12 treatment groups compared with those in the D-gal group (Figures 7(a)–7(c)). This result further suggested that Bifidobacteria BB-12 can promote the macrophage M2-polarized change, and the STAT6 may be the main mediated factor.

## 4. Discussion

Immunosenescence, which is an important change in the aging of organism, facilitates the genesis and development of age-related diseases, such as Alzheimer's disease [41] and tumor [42]. Immune cell aging is the basic change in immunosenescence, which is specifically manifested by the declining quantity of immune cells, damaged immunoreaction capacity, and weakened intercellular coordination ability [43, 44]. The researchers found that macrophages can also experience change due to aging with organism aging [45]. Oxidative stress level in senescent macrophages is significantly increased compared with that in young macrophages, thereby indicating that ROS is one of factors facilitating macrophage aging [46]. According to our previous research [9, 10], D-gal, which is a ROS promoter, was first used to induce macrophage aging in vitro. As D-gal concentration increases, the quantity of SA-*β*-gal-stained-positive J774A.1 macrophages gradually increased, the expression levels of aging-related proteins p53 and p16 increased, the cell proliferation ability was weakened, and the intracellular ROS level gradually increased. These results indicated that D-gal can induce macrophage aging. The 1.0 and 10.0 g/L groups had evident facilitating effects on macrophage aging.

The two dominant states of macrophage activation were described as follows: M1 or M2 under different environmental stimulation conditions [47]. Aging macrophage can shift differently from anti-inflammatory M2 into proinflammatory M1. In aging mice, the expression levels of the M1 macrophage markers CD68, tumor necrosis factor-*α*, and IL-6 increased, and those of the M2 macrophage markers CD36 and IL-10 decreased [6]. However, the inhibition of cellular senescence can facilitate macrophage differentiation into M2 state and upregulate the expression levels of the M2 macrophage markers, such as Arg-1 [7], MRC1, and AMAC1 [8]. In the present study, with the increase in macrophage aging degree, the secreted amounts of the M1 markers IL-6 and IL-12 gradually increased, while the M2 marker IL-10 and Arg-1 mRNA levels gradually decreased. These results showed that aging macrophage can be differentiated into proinflammatory M1 type, which exerted an important effect on inflamm-aging.

Bifidobacterium is an important intestinal probiotic [48, 49]. The recent study indicated that Bifidobacterium can significantly increase the longevity of *Caenorhabditis elegans* [50, 51]. The present study showed for the first time that Bifidobacterium BB-12 can considerably delay J774A.1 macrophage aging. As the treatment time passed by, the quantity of SA-*β*-gal-positive cells in the BB-12 treatment group gradually reduced, and expression levels of the aging-related proteins p53 and p16 decreased. Thus, BB-12 had an evident protective effect on D-gal-induced macrophage aging. Further polarity detection results showed that BB-12 supernatant treatment can reverse the inductive effect of D-gal on macrophage M1 polarization and promote the expression of J774A.1 macrophage M2 polarization marker IL-10 and Arg-1. STAT1 and STAT6 are the key molecules mediating polar differentiation of macrophages M1 and M2 [52, 53] and closely related to immunosenescence [54, 55]. To confirm the influence of Bifidobacterium on M1 and M2 polarization further, we further detected the influences of BB-12 on STAT1 and STAT6. As the BB-12 treatment time continued, p-STAT1 expression gradually reduced, while p-STAT6 expression gradually increased. The results further verified that BB-12 can inhibit the differentiation of macrophages into M1 type and facilitate M2 macrophage polarization, thereby indicating that BB-12 can resist inflammatory aging. Burns et al. [56] also found that BB-12 can relieve colonic inflammatory state in mice, which was consistent with the experimental results in the present study.

## 5. Conclusion

The results of this study showed that D-gal can induce J774A.1 macrophage aging, increase the expression levels of aging-related proteins p53 and p16, and facilitate J774A.1 macrophage polarization into M1 type by promoting ROS generation. Bifidobacterium BB-12 can reverse the inductive effect of D-gal on J774A.1 macrophage aging and result in J774A.1 macrophage polarization into M2 type by promoting p-STAT6 expression. This result indicated that Bifidobacterium can delay immunosenescence and inflamm-aging. The effective constituents of Bifidobacterium, which regulates macrophage aging and their molecular mechanism, should be further studied and explored.

## Data Availability

The data used to support the findings of this study are available from the corresponding author upon request.

## Supplementary Materials

Supplementary Materials Supplementary Figure 1: the results of SA-*β*-gal staining showed that D-gal can induce peritoneal macrophage senescence. Supplementary Figure 2: the results of SA-*β*-gal staining showed that BB-12 treatment can remarkably reverse the induced role of D-gal on peritoneal macrophage senescence. Supplementary Figure 3: BB-12 can lead to a shift in peritoneal macrophage cells from the M1-polarized state to the M2-polarized state.
