# Supplementary material for: Bifidobacterium lactis BB-12 Attenuates Macrophage Aging Induced by D-Galactose and Promotes M2 Macrophage Polarization
Source: J Immunol Res. 2019 Dec 19;2019:4657928. doi: 10.1155/2019/4657928 (PMC6942849; doi:10.1155/2019/4657928)
Supplement: Supplementary Materials — Supplementary Figure 1: the results of SA-β-gal staining showed that D-gal can induce peritoneal macrophage senescence. Supplementary Figure 2: the results of SA-β-gal staining showed that BB-12 treatment can remarkably reverse the induced role of D-gal on peritoneal macrophage senescence. Supplementary Figure 3: BB-12 can lead to a shift in peritoneal macrophage cells from the M1-polarized state to the M2-polarized state. [file 4657928.f1.docx]

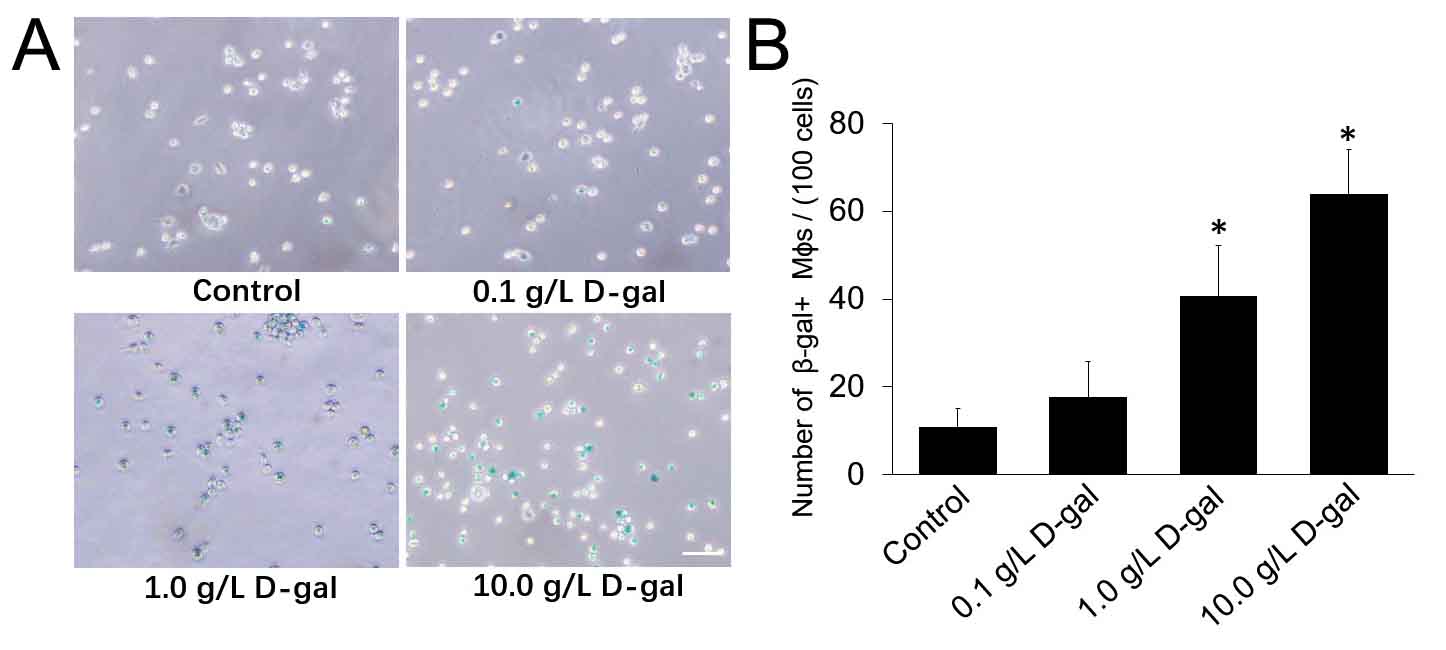


**Figure S1.** Effects of D-gal on peritoneal macrophages senescence. (A) SA-𝛽-gal staining. To obtain peritoneal macrophages, the SD rats (3 months) were intraperitoneally injected with 10 ml of thioglycollate. Four days later the animals were sacrificed and peritoneal exudate cells were harvested by peritoneal lavage with 10 mL of icecold PBS and peritoneal macrophages were purified using a macrophages enrichment kit (Xuanya Biotechnology, Shanghai, China) as per manufacturer’s instructions. After treated by different concentration D-gal for 24 h, SA-𝛽-gal staining shown that the number of SA-𝛽-gal positive cells increase in the 1.0 and 10.0 D-gal group. Scale bar = 50 𝜇m. (B)Quantification of SA-𝛽-gal–staining cells. The total number of SA-𝛽-gal-positive cells among 100 random cells was counted using phase-contrast microscopy. The results showed that the number of SA-𝛽-gal-positive peritoneal macrophages /100 cells in the 1.0 and 10.0 D-gal groups was markedly increased than that in the control group (* *P*＜0.01,n=5).


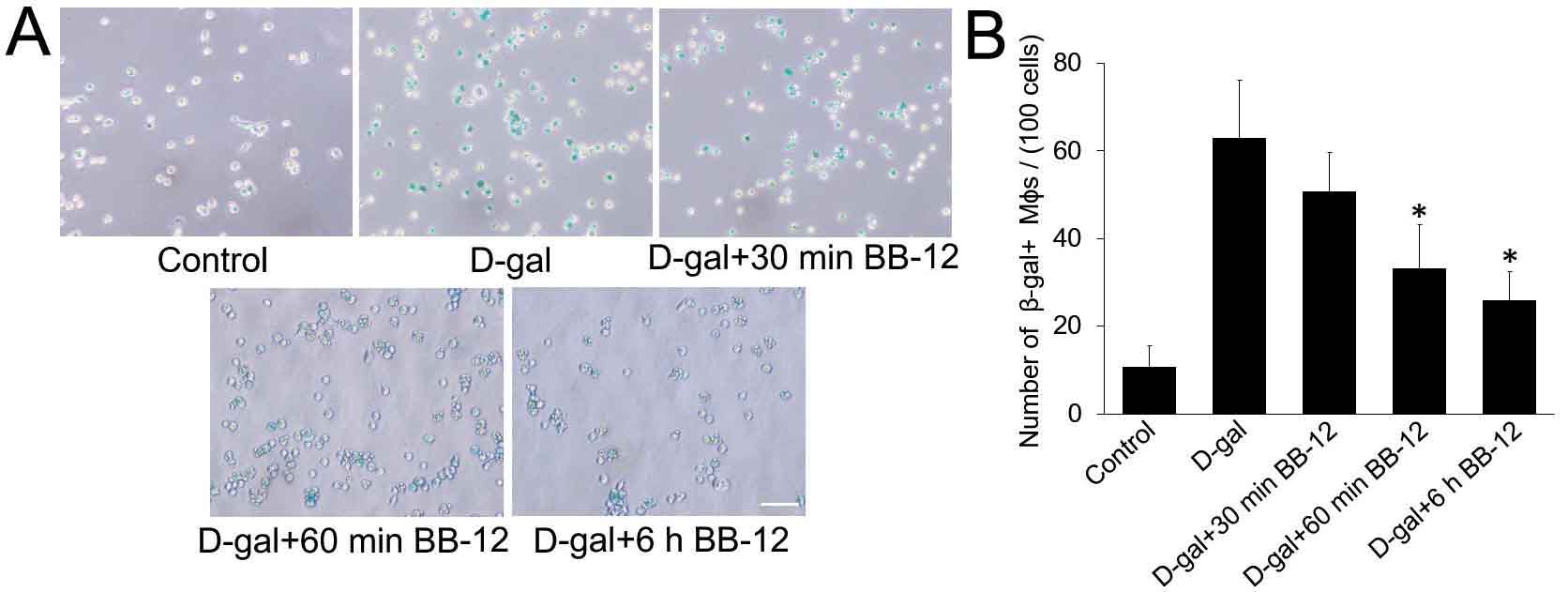


**Figure S2.** Effects of BB-12 on peritoneal macrophages senescence: (a) SA-β-gal staining. Compared with the D-gal group, after treatment with BB-12 for different time, the SA-β-gal positive cells in the BB-12 treatment group were clearly decreased. (b)Quantification of SA-β-gal-positive cells. The counts showed that the number of SA-β-gal positive peritoneal macrophages in the D-gal +60min BB-12 and D-gal +6h BB-12 groups decreased significantly compared with that in the D-gal group(**P*＜0.01, n=5).


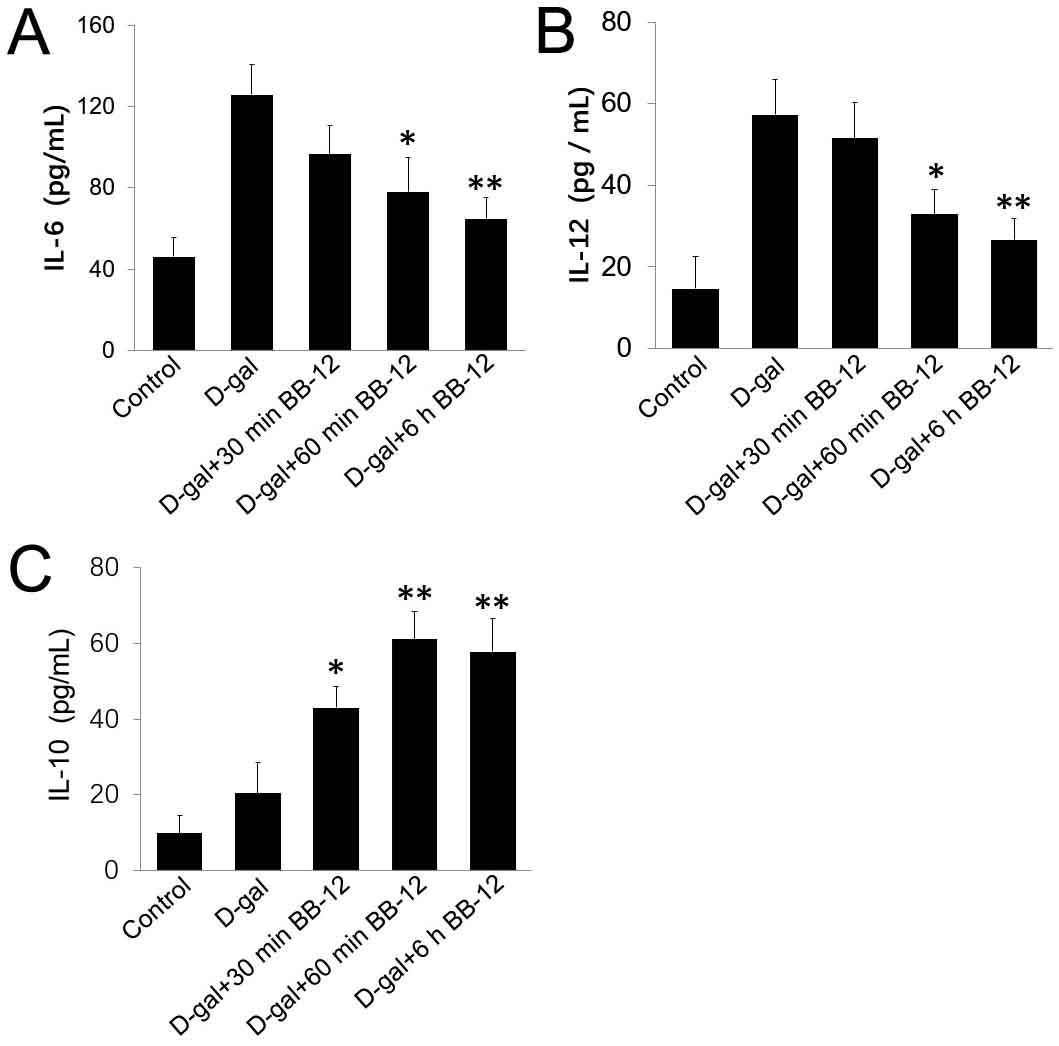


**Figure S3.** Effects of BB-12 on the polarization of peritoneal macrophage. The production of IL-6 (A), IL-12 (B) and IL-10 (C) by were assayed by ELISA. The results showed that IL-6 and IL-12 level in the D-gal+60min group and D-gal+6h group were clearly decreased compared with that in

the D-gal group (**P*＜0.05 or ***P*＜0.01, n=3). IL-10 in the D-gal+30min group, D-gal+60min group and D-gal+6h group were significantly higher than that in the D-gal group (**P*＜0.05 or ***P*＜0.01, n=3). The results showed that BB-12 could repolarize M1 phenotype cells toward M2 phenotype.
